# *In Vivo* Removal of N-Terminal Fusion Domains From Recombinant Target Proteins Produced in *Nicotiana benthamiana*

**DOI:** 10.3389/fpls.2020.00440

**Published:** 2020-04-08

**Authors:** Md Reyazul Islam, Seoyoung Choi, Thangarasu Muthamilselvan, Kunyoo Shin, Inhwan Hwang

**Affiliations:** ^1^ Division of Integrative Biosciences and Biotechnology, Pohang University of Science and Technology, Pohang, South Korea; ^2^ Department of Life Sciences, Pohang University of Science and Technology, Pohang, South, Korea

**Keywords:** *Nicotiana benthamiana*, human leukemia inhibitory factor, plant-based expression systems, small ubiquitin-related modifier, small ubiquitin-related modifier-specific protease, mouse embryonic stem cells

## Abstract

Plants show great potential for producing recombinant proteins in a cost-effective manner. Many strategies have therefore been employed to express high levels of recombinant proteins in plants. Although foreign domains are fused to target proteins for high expression or as an affinity tag for purification, the retention of foreign domains on a target protein may be undesirable, especially for biomedical purposes. Thus, their removal is often crucial at a certain time point after translation. Here, we developed a new strategy to produce target proteins without foreign domains. This involved *in vivo* removal of foreign domains fused to the N-terminus by the small ubiquitin-related modifier (SUMO) domain/SUMO-specific protease system. This strategy was tested successfully by generating a recombinant gene, *BiP:p38:bdSUMO : His:hLIF*, that produced human leukemia inhibitory factor (hLIF) fused to p38, a coat protein of the *Turnip crinkle virus*; the inclusion of p38 increased levels of protein expression. The recombinant protein was expressed at high levels in the leaf tissue of *Nicotiana benthamiana*. Coexpression of bdSENP1, a SUMO-specific protease, proteolytically released His:hLIF from the full-length recombinant protein in the endoplasmic reticulum of *N. benthamiana* leaf cells. His:hLIF was purified from leaf extracts *via* Ni^2+^–NTA affinity purification resulting in a yield of 32.49 mg/kg, and the N-terminal 5-residues were verified by amino acid sequencing. Plant-produced His:hLIF was able to maintain the pluripotency of mouse embryonic stem cells. This technique thus provides a novel method of removing foreign domains from a target protein *in planta*.

## Introduction

Recombinant proteins have a wide range of uses from biomedical applications to industrial purposes. Across this range of applications, a crucial question is how functional proteins may be produced on a large scale and at an affordable cost. Many different organisms, including bacteria, fungi, cells of insects and other animals, and plants, have been developed as platforms for producing recombinant proteins. Although the bacterial system was the first to be developed, animal cell cultures are now the most widely used systems for producing the recombinant proteins used in protein drugs. The advantages and disadvantages of the different systems depend on the type of recombinant protein under production (Tripathi, 2016; Bielser et al., 2018; Hitzeman et al., 2018; Owczarek et al., 2019; Schillberg et al., 2019).

Recently, rapid advances have been made in the development of plant platforms for recombinant protein production (Takeyama et al., 2015; Yao et al., 2015; Schillberg et al., 2019). A major focus of research has been on how best to express high levels of recombinant proteins in plants. One means of achieving this goal involves the chloroplast gene expression system, in which foreign genes are integrated into the chloroplast genome *via* homologous recombination (Zhang et al., 2017). This approach enables the high expression of a target gene *via* target gene amplification, a process that involves the two different mechanisms that generate a high number of chloroplasts per cell and a high copy number of the chloroplast genome per chloroplast. High-level expression of foreign genes has also been achieved *via* nuclear gene expression systems, which use plant viruses-derived vectors (Twyman et al., 2002). Vectors derived from DNA or RNA viruses are able to amplify DNA or mRNA, respectively, leading to high-level expression of foreign genes (Gleba et al., 2005; Yamamoto et al., 2018).

A completely different approach has involved the identification of particular domains that, when fused to a target protein, increase expression levels; for example, elastin-like polypeptides (ELP), protein–polymers composed of pentapeptide repeat sequence (VGVPG)_(5)_ that induce endoplasmic reticulum (ER)-derived protein bodies, when fused to a target protein significantly improve production yield in plants (Conley et al., 2011). Moreover, the fusion of fungal hydrophobins (HFBI) (Gutiérrez et al., 2013) and the proline-rich domain of *γ*-zein (Conley et al., 2011), a maize seed storage protein, increase the accumulation of recombinant proteins in plants. Fusions of an N-glycosylation domain (M domain) derived from human CD45 to either the C- or N-terminus (Kang et al., 2018), and of SBA, a sugar-binding lectin from soybean (Alqazlan et al., 2019), have also recently been shown to greatly enhance expression of fusion proteins in plants.

Another critical issue in the development of plant platforms is purification of the recombinant proteins from plant extracts. One efficient means of protein purification involves the use of an affinity tag. These tags are commonly fused to a target protein, allowing affinity-based purification (Arnau et al., 2006). Several affinity tags have been developed for purifying proteins from plant extracts, including the poly-histidine (His×6) tag, the cellulose-binding domain (CBD), and the crystallizable fragment (F_C_) antibody region.

Fusion of any of these foreign domains to a target protein inevitably alters the nature of that protein, and thus their presence is often undesirable, especially on recombinant proteins intended for biomedical use. It is frequently necessary to remove the domains following expression and purification of the target protein. The techniques developed to remove a foreign domain or tag from a protein include *in vitro* proteolysis using proteases such as enterokinase (EK), thrombin Xa, and *tobacco etch virus* (TEV) protease (Arnau et al., 2006). Each of these enzymes recognizes a specific short peptide sequence and cleaves a specific site within or at the end of the recognition sequence; a specific proteolytic cleavage site can thus be incorporated between the target protein and the foreign domain or tag. As most proteases cleave either at the C-terminal end or on the C-terminal side of their specific recognition sequences, tag-less recombinant proteins are obtained by fusing either a foreign domain to ensure high-level expression or a purification tag to the N-terminus of the target protein.

In addition to such well-established enzymes, a highly active proteolytic enzyme, SUMO-specific protease, derived from *Brachypodium distachyon* (*B. distachyon*) has recently been used to remove a foreign domain *in vitro* (Frey & Görlich, 2014; Islam et al., 2019a). This protease recognizes the entire SUMO domain (Bailey and O’Hare, 2004; Hickey et al., 2012), and thus there is no possibility of nonspecific cleavage of the protein substrate. Moreover, SUMO-specific protease leaves no extra residues on the target protein after cleavage (Hickey et al., 2012; Islam et al., 2019a).

We developed an *in vivo* proteolytic cleavage system that used the SUMO domain and SUMO-specific protease (bdSENP1) to remove foreign domains from a target protein in plants (*Nicotiana benthamiana*). Our chosen target protein was human leukemia inhibitory factor (hLIF), a multifunctional cytokine that is a member of the interleukin 6 (IL-6) family (Hirai et al., 2011). LIF activates the JAK-STAT3 signaling pathway, and thereby plays a key regulatory role in maintaining the pluripotent state of embryonic stem cells (ESCs) through suppression of stem cell differentiation (Shabbir et al., 2010; Hirai et al., 2011). We found that the N-terminal fusion of p38, the coat protein of *Turnip crinkle virus*, to His:hLIF led to high-level expression in *N. benthamiana*. Moreover, the N-terminal foreign domain p38, together with the SUMO domain, was efficiently removed from the C-terminal of His:hLIF by coexpression of bdSENP1 in the ER of plant cells. Purified His:hLIF from *N. benthamiana* leaf tissue extract was biologically active and maintained the pluripotency of mouse embryonic stem cells (mESCs).

## Materials and Methods

### Construction of Plant Expression Vectors

The small cellulose-binding domain (*sCBD*) derived from *Trichoderma reesei* (AEP40512.1), *SUMO* domain (amino acids 21-97) of bdSUMO (XP_003564931.1), *bdSENP1* (catalytic region; amino acid positions 242-481, XP_003567671.1), green fluorescent protein (*GFP*), *hIL6* (mature region from amino acid positions 30-212, P05231), *hLIF* (active region from amino acid positions 23-202, P15018), and coat protein *p38* (P06663) were codon-optimized based on the codon usage of *N. benthamiana* and were chemically synthesized (Bioneer corp., Daejeon, Korea).

**Figure 1:**
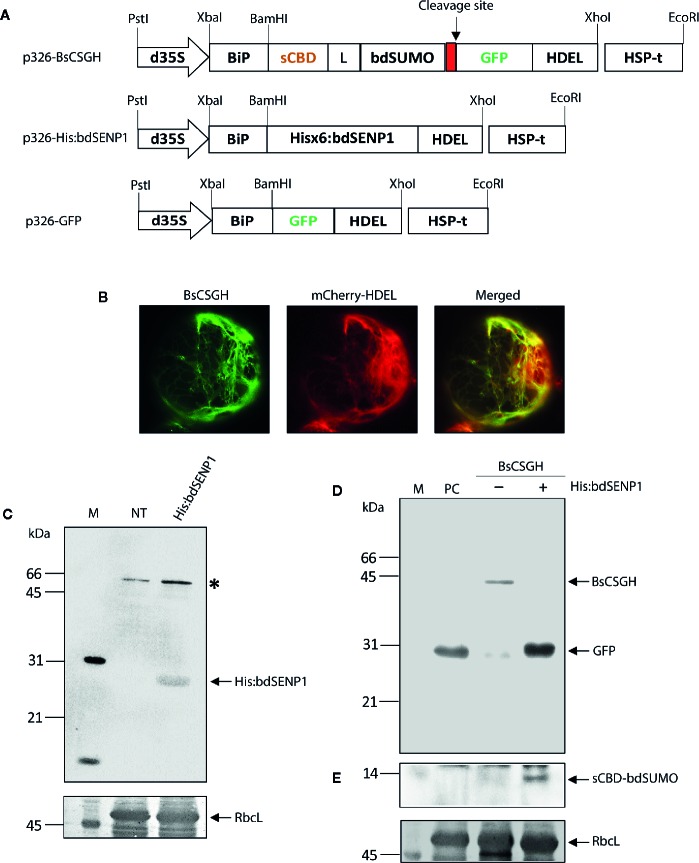
*In vivo* cleavage of bdSUMO domain-containing proteins by bdSENP1 coexpressed in the endoplasmic reticulum of *Arabidopsis thaliana* protoplasts. **(A)** Schematic representation of constructs used in *Arabidopsis thaliana* protoplasts. sCBD, cellulose-binding domain, derived from *Trichoderma reesei*; bdSUMO, small ubiquitin-related modifier of *B. distachyon* (XP_003564931.1); bdSENP1, SUMO-specific protease (XP_003567671.1) of *B. distachyon*; GFP, green fluorescent protein. All expression constructs were under the control of the cauliflower mosaic virus (CaMV) 35S promoter with the double-enhanced element (d35S) and the HSP terminator (HSP-t) of *A. thaliana*. The endoplasmic reticulum (ER) leader sequence, BiP, and an ER retention signal, HDEL, were fused to the 5′ and 3′ ends of the chimeric constructs, respectively. **(B)** Localization of BsCSGH in the ER of *A. thaliana* protoplasts. Protoplasts were transformed with *p326-BsCSGH*, and subcellular localization of BsCSGH was observed using fluorescence microscopy. BiP:mCherry : HDEL was coexpressed as a marker for the ER. Left panel: BsCSGH (green); middle panel: BiP:mCherry : HDEL (red); right panel: merged image. **(C)** Western blot analysis of His:bdSENP1. Total soluble proteins were isolated from *A. thaliana* protoplasts transformed with *p326-His:bdSENP1* and from nontransformed control protoplasts (NT) and analyzed by western blotting with an anti-His antibody. The membrane was subsequently stained with Coomassie brilliant blue (CBB). Asterisk (*) indicates nonspecific bands. **(D**, **E)** Cleavage in the ER of recombinant protein containing the bdSUMO domain by coexpressed His:bdSENP1. *A. thaliana* protoplasts were transformed with *p326-BsCSGH*, alone (−) or together with *p326-His:bdSENP1* (+). Total soluble proteins were extracted from transformed protoplasts and analyzed by western blotting with an anti-GFP antibody **(D)** or the same membrane subsequently stripped of antibody and reprobed with anti-CBD antibody **(E)**. Membranes were stained with CBB after immunoblotting. p326-GFP was used as a positive control (PC). The large subunit of the rubisco complex (RbcL) was used as a loading control. The arrows indicate BsCSGH at 42 kDa; GFP at 29 kDa; and sCBD-bdSUMO at 13 kDa. M, molecular weight standard.

The fusion constructs *sCBD:bdSUMO : GFP* and *sCBD:bdSUMO:hIL6* were generated by overlap PCR using primers sCF/suR/GF/GR, and sCF/suILR/ILF/ILR (**Supplementary Table S1**), respectively. The final PCR products were digested with *BamHI* and *XhoI* restriction endonucleases and introduced into the 326-plant expression vector (Kim et al., 2013) to give p326-sCBD:bdSUMO : GFP (p326-BsCSGH) (**Figure 1A**) and p326-sCBD:bdSUMO:hIL6 (p326-BsChIL6H) (**Supplementary Figure S1A**). *His:bdSENP1* and *bdSENP1:HA* for expression in plants were generated by PCR amplification using the primer sets HisSF-1/SR-2 and SF-3/SHaR-4 (**Supplementary Table S1**), respectively. The PCR products were digested with *BamHI* and *XhoI* restriction endonucleases and introduced into the plant expression vector, 326-GFP which had been digested with the same restriction endonucleases, to give p326-His:bdSENP1 (**Figure 1A**) and p326-bdSENP1:HA. The synthetic *hLIF* gene was incorporated to the C-terminus of M:CBM3:bdSUMO by overlapping PCR using primers csLF-1, csLR-2, csLF-3, and csLR-4 (**Supplementary Table S1**) using 326-M:CBM3:bdSUMO:hIL6 as a template (Islam et al., 2019a). Six histidine residues (His) were added at the N-terminus of hLIF (*His:hLIF*) during PCR amplification. The final overlapping PCR product, *M:CBM3:bdSUMO : His:hLIF* (*MCS:hLIF*), and PCR amplified *His:hLIF* were digested with *BamHI* and *XhoI* restriction endonucleases and introduced at the same site in the plant expression vector, 326-M:CBM3:bdSUMO:hIL6 to give 326-MCS:hLIF and 326-His:hLIF, respectively. The synthetic *p38* gene was PCR amplified using the primer set PF-1 and PR-2 (**Supplementary Table S1**) and ligated into 326-MCS:hLIF vector after digestion with *BamHI* and *XmaI* restriction endonucleases to generate the 326-p38:bdSUMO:hLIF. An ER retention signal, His-Asp-Glu-Leu (HDEL) (Gomord et al., 1997), was also added to the C-terminus of the coding sequence of all expression constructs during PCR amplification.

**Figure 2:**
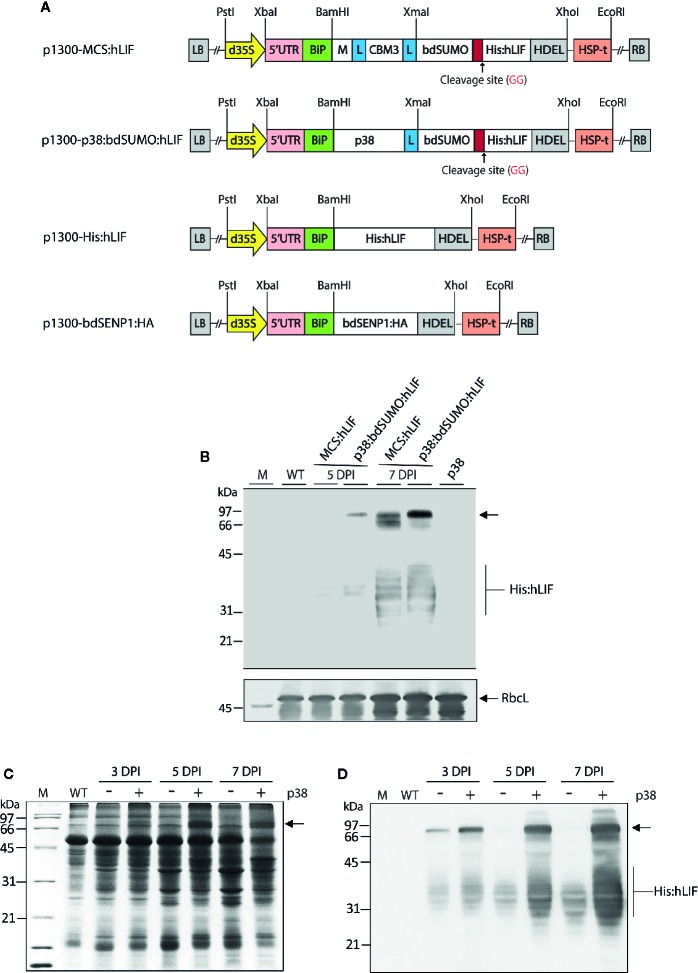
*Agrobacterium tumefaciens*-mediated transient expression of MCS:hLIF and p38:bdSUMO:hLIF in *Nicotiana benthamiana.* **(A)** Schematic showing the chimeric binary constructs for *Agrobacterium tumefaciens*-mediated transient expression in *Nicotiana benthamiana* leaf tissues. p1300-MCS:hLIF, p1300-p38:bdSUMO:hLIF, p1300-His:hLIF, and p1300-bdSENP1:HA are binary vectors for protein expression in plants. The double enhancer-containing CaMV 35S promoter (d35S) and the HSP terminator (HSP-t) from *A. thaliana* were used as promoter and terminator, respectively. LB, left border; RB, right border. **(B)** Western blot analysis of MCS:hLIF and p38:bdSUMO:hLIF expression. *N. benthamiana* leaf tissue was harvested 5 and 7 days post infiltration (DPI) with *A. tumefaciens*-infiltration of the chimeric recombinant genes with *p38*. Total extracts were prepared and analyzed by western blotting with anti-LIF antibody. Wild-type *N. benthamiana* (WT) and p38-infiltrated leaf extracts were loaded as negative controls. The membrane was subsequently stained with Coomassie brilliant blue (CBB). The large subunit of the rubisco complex (RbcL) was used as a loading control. **(C**, **D)** Expression levels of p38:bdSUMO:hLIF at different time points after *A. tumefaciens*-infiltration. *N. benthamiana* leaves were infiltrated with *A. tumefaciens* harboring *p1300-p38:bdSUMO:hLIF*, either with (+) or without (−) an *Agrobacterium* culture harboring *p1300-p38* of the *Turnip crinkle virus* silencing suppressor. Leaves were harvested at 3 and 5 DPI, and total leaf extracts were separated by 12.5% SDS-PAGE followed by CBB-staining **(C)** or western blot analysis with anti-LIF antibody **(D)**. The arrows indicate the positions of p38:bdSUMO:hLIF and MCS:hLIF protein bands at 66 and 97 kDa, respectively, and His:hLIF at 30 to 42 kDa. M, molecular weight standard.

To binary expression constructs, *326-MCS:hLIF*, *326-His:hLIF*, *326-p38:bdSUMO:hLIF*, and *326-bdSENP1:HA* constructs were digested with *PstI* and *EcoRI* restriction endonucleases and ligated to a binary construct *p1300-C3bdSU-hIL6* (Islam et al., 2019a) digested with the same restriction endonucleases to give p1300-MCS:hLIF, p1300-His:hLIF, p1300-p38:bdSUMO:hLIF and p1300-bdSENP1:HA, respectively (**Figure 2A**). For the expression of genes in plants, cauliflower mosaic virus (CaMV) 35S promoter with the double enhancer (d35S), 5′ untranslated enhancer region (5′ UTR) (Kim et al., 2013), the BiP leader sequence (BAA13947; amino acid positions 1 to 34), and the HSP terminator from *Arabidopsis thaliana* (Nagaya et al., 2010) were used. The nucleotide sequences of all constructs were confirmed by DNA sequencing (Macrogen Inc., Seoul, Korea).

### Transient Expression in *Arabidopsis* and *N. benthamiana*

Plasmid DNA of p326-BsCSGH, p326-BsChIL6H, and p326-His:bdSENP1 was isolated by the Qiagen plasmid DNA isolation kit (Qiagen, CA, USA) and used for transformation into *Arabidopsis* protoplasts by polyethylene glycol (PEG)-mediated transformation (Yoo et al., 2007).

The binary plant expression vectors p1300-MCS:hLIF, p1300-His:hLIF, p1300-p38:bdSUMO:hLIF, and p1300-bdSENP1:HA were transformed into *Agrobacterium tumefaciens* (*A. tumefaciens)* EHA105 strain by electroporation. *A. tumefaciens* EHA 105 carrying p1300-MCS:hLIF, p1300-His:hLIF, p1300-p38:bdSUMO:hLIF, or p1300-bdSENP1:HA, or *A. tumefaciens* harboring p1300-p38 of the *Turnip crinkle virus* silencing suppressor (**Supplementary Figure S5**) (Qu et al., 2003) were cultured in YEP liquid medium consisting of 1% yeast extract, 1% tryptone peptone, 0.5% NaCl (pH 7.0), supplemented with 50 µg/ml kanamycin and 50 µg/ml rifampicin at 27~28°C for 24–36 h by constant shaking at 200 rpm. *A. tumefaciens* cells were pelleted by centrifugation (3,500 × g for 10 min) and resuspended in the infiltration buffer (pH 5.6) containing 10 mM MgCl_2_, 10 mM MES, and 200 µM acetosyringone at 0.8 of OD_600_. For coexpression of multiple genes, two or three bacterial suspension cultures were mixed at a 1:1 ratio (v/v). *N. benthamiana* leaf tissues (5 to 7-week-old) were infiltrated with 1 ml syringe without a needle or either using the vacuum infiltration system with the mixture of *A. tumefaciens* cultures (Marillonnet et al., 2005). Subsequently, plants were further grown for 3 to 7-days post infiltration (DPI) in the greenhouse under the standard conditions.

To prepare total soluble protein (TSP), infiltrated *N. benthamiana* leaf tissues were ground to a fine powder in liquid nitrogen and homogenized in protein extraction buffer (50 mM Tris-HCl, pH 7.5, 150 mM NaCl, 1 mM DTT, 0.1% [v/v] Triton X-100 and protease inhibitor cocktail). TSP was clarified by centrifugation (13,000 × g) for 20 min, and protein concentrations were determined by the Bradford method (Bradford, 1976) (Bio-Rad, Hercules, CA, USA).

### SDS-PAGE and Western Blot Analysis

TSP or purified proteins were separated using 10–12.5% sodium dodecyl sulfate polyacrylamide gel electrophoresis (SDS-PAGE) under the reducing condition, and the proteins on the gels were either stained with 0.25% Coomassie blue R-250 (AMRESCO, cat. no: 6104-59-2, Zottegem, Belgium) solution or analyzed by Western blotting using mouse anti-His (Qiagen, Valenica, CA, USA), mouse anti-LIF (abcam, cat. no., ab34427, Cambridge, UK), mouse anti-GFP (Clontech, cat. No., 632381, CA, USA), rat anti-HA (Roche, cat. No., 1583 816, Basel, Switzerland), mouse anti-IL6 (abcam, cat. no., ab9324, Cambridge, UK), and anti-CBD (Bioapp, Pohang, Korea) antibodies. The horseradish peroxidase (HRP)-conjugated sheep anti-mouse IgG (Bethyl, cat. no., A90-146P, TX, USA), and goat anti-rat IgG (Bethyl, cat. no., A110-105P, TX, USA) were used as secondary antibody. Bands on the immunoblots were detected using chemiluminescence detection reagents (GE healthcare, Illinois, USA), and images were captured with a LAS 4000 image capture system (Fujifilm, Tokyo, Japan).

### Ni^2+^–NTA Affinity Protein Purification

TSP containing His-tagged hLIF was clarified by centrifugation (15,000g × 30 min) at 4°C. His:hLIF was purified using an Ni^2+^–NTA agarose column (Qiagen, CA, USA) according to the manufacturer’s instructions. Finally, eluted fractions containing His:hLIF were concentrated using a 10 K centricon centrifugal filter (Millipore, cat. no. 4304, Darmstadt, Germany). During the concentration process, the elution solution was replaced with PBS buffer.

### N-Terminal Amino Acid Sequencing

Purified His:hLIF (5 µg) was separated *via* 12% SDS-PAGE under the reducing condition and electroblotted onto polyvinylidene difluoride (PVDF) membrane (Millipore, Darmstadt, Germany). The PVDF membrane was stained with 0.02% Coomassie Blue R-250 until protein bands were visualized clearly. Proteins between 30 and 42 kDa bound to PVDF membrane were subjected to Edman degradation using an ABI 492 Procise Protein Sequencer System (Applied Biosystems, CA, USA).

### Enzymatic Deglycosylation of the Plant-Produced His:hLIF

To remove the N-glycan moiety of His:hLIF, purified His:hLIF was treated with endo-*β*-N-acetylglucosaminidase H (Roche, Cat. No. 11088726001, Basel, Switzerland). Briefly, 2 µg of purified hLIF was denatured using glycoprotein denaturing buffer (0.5% SDS and 1.0% *β*-ME) at 100°C for 10 min and then treated with 1 µl (0.005 U) of Endo-H at 37°C for 4 h.

### Determination of Endotoxin Content

The level of endotoxin in the purified hLIF was determined by Pierce’s LAL Chromogenic Endotoxin quantitation kit (Thermo Fisher Scientific, Cat. no. 88282, MA USA). The three biological replicates were used for endotoxin determination. The standard curve was generated using a commercial *E. coli* endotoxin standard provided by the kit manufacturer.

### Mouse Embryonic Stem Cells Culture

Mouse embryonic stem cells (mESCs) (ATCC, SCRC-1002) were maintained on mitomycin C-treated MEF feeder cells. Mitotically inactivated MEFs were seeded at a density of 1.1 × 10^5^ cells per 12-well plate, and the next day, mESCs were seeded at a density of 1.4 × 10^5^ cells per 12-well plate. mESCs were incubated in DMEM (Thermo Fisher Scientific, MA, USA) containing 200 mM Ala-Gln (Sigma-Aldrich, Missouri, USA), 1× minimum essential media-nonessential amino acids (Thermo Fisher Scientific, MA USA), 100 µM 2-mercaptoethanol (Thermo Fisher Scientific, MA USA), 1% penicillin/streptomycin (Thermo Fisher Scientific, MA USA), 15% FBS (Millipore, Darmstadt, Germany), 10 ng/ml commercial LIF (Merck Millipore, MA, USA), or 10 ng/ml His:hLIF (phLIF) derived from plant. The medium was changed every day, and mouse mESCs were passaged every 2 days.

### Quantitative RT-PCR

Dissociated mouse mESCs were plated on the gelatin-coated dish and incubated at 37°C for 15 min to deplete feeder cells. Total RNA was extracted from feeder-depleted mouse ESCs using RNeasy Mini Kit (Qiagen, CA, USA). Total RNA (2 μg) was used to prepare cDNA using high capacity cDNA Reverse Transcription Kit (Applied Biosystems, CA, USA). qRT-PCR was performed on a 7500 ABI Real-time PCR system (Applied Biosystems, CA, USA) using the Power SYBR Green PCR master mix (Applied Biosystems, CA, USA) to check the expression levels of pluripotent marker genes *Oct4*, *Sox2*, and *Nanog*. Gene expression was normalized to the housekeeping gene *Hprt*. The PCR mixture (20 μl) contained 200 ng of template, 0.5 μM forward and reverse primers, and 1× SYBR master mix. The PCR conditions were as follows: initial denaturation at 95°C for 10 min, followed by 40 cycles of 95°C for 15 s and 60°C for 1 min. To confirm specific amplification, a melting curve was generated by heating at 95°C for 15 s and then at 60°C for 1 min and then increasing the temperature by 0.3°C every 15 s up to 95°C. All primers are listed in **Supplementary Table S2**. The data were collected from six independent experiments, and the technical replicates were repeated three times. Statistical analysis was performed by one-way analysis of variance (ANOVA) with Tukey’s *post-hoc* multiple comparison analysis (GraphPad Prism 6).

## Results

### bdSENP1 Cleaves the bdSUMO Cleavage Site in Recombinant Protein in the ER

The SUMO-specific protease (bdSENP1) from *B. distachyon* was selected to serve as the basis for a protocol for the removal of foreign domains from target proteins *in vivo*. This protease is highly active and leaves no extra residues attached to the target protein after cleavage. Although bdSENP1 functions in the cytoplasm (Bailey and O’Hare, 2004; Hickey et al., 2012), we intended target proteins to accumulate in the ER. The ER localization renders recombinant proteins to be subject to posttranslational modification such as N-glycosylation and disulfide bond formation, which are critical for protein folding, stability, and functionality (Gomord and Faye, 2004). In addition, targeting of recombinant proteins to the ER significantly improved production yields in plants (Schouten et al., 1996; Nausch et al., 2012; Kang et al., 2018). Therefore, we first determined whether bdSENP1 was capable of cleaving its substrate in the ER *in vivo.* We generated a bdSENP1 expression construct containing a His×6 tag, *His:bdSENP1* (*BiP : His:bdSENP1:HDEL*) (**Figure 1A**) and a bdSENP1 substrate construct, *BsCSGH* (*BiP:sCBD:bdSUMO : GFP:HDEL*), that incorporated the leader sequence of an ER chaperone BiP, a small cellulose-binding domain (sCBD) derived from *Trichoderma reesei*, the bdSUMO domain from *B. distachyon*, and green fluorescent protein (GFP) tagged with HDEL, an ER retention sequence (**Figure 1A**).

We used PEG-mediated transformation (Yoo et al., 2007) to examine bdSENP1 activity in the ER in *Arabidopsis thaliana* (*A. thaliana*) protoplasts. Protoplasts were transformed with either *BsCSGH* alone or *BsCSGH* plus *His:bdSENP1*; in addition, protoplasts were transformed with *BiP : GFP:HDEL*, which expressed GFP alone in the ER lumen, as a positive control (PC). A fluorescence microscope was used to determine the expression and subcellular localization of BsCSGH. Protoplasts transformed with *BsCSGH* displayed strong GFP signals in a net-like pattern, an indication of ER localization (**Figure 1B**), strongly suggesting that BsCSGH was targeted to this organelle. To determine the expression at the biochemical level, total soluble proteins were extracted from protoplasts and analyzed by western blotting with an anti-GFP antibody (**Figure 1D**); extracts from untransformed protoplasts were included as a negative control. A 42 kDa band was detected by the anti-GFP antibody in extracts in which BsCSGH alone was expressed, indicating a BsCSGH-specific protein band; by contrast, when *His:bdSENP1* was coexpressed with *BsCSGH*, a 29 kDa protein was detected that was the same size as the GFP used as the control. These results indicate that bdSENP1 could digest the BsCSGH proteolytically by recognizing the bdSUMO domain when both were expressed in the ER (**Figure 1D**). Moreover, western blot analysis of protein extracts using anti-CBD antibody detected a 13 kDa band, corresponding to the sCBD:bdSUMO fragment (**Figure 1E**), further confirming the proteolytic cleavage of BsCSGH by bdSENP1 in the ER. Expression of His:bdSENP1 in the protoplasts was confirmed by western blotting with anti-His antibody (**Figure 1C**); this detected a 28 kDa protein band at the predicted position for His:bdSENP1. These findings suggested that when bdSENP1 was localized to the ER, it recognized the bdSUMO domain and cleaved the protein at the C-terminus of bdSUMO cleavage site.

To further validate this finding, we used another chimeric construct to express human interleukin-6 (hIL6) as the target protein. hIL6 is a cytokine and plays an important function as both a proinflammatory and an anti-inflammatory (Kishimoto, 2010). We previously used a chimeric protein (*BiP:M:CBM3:bdSUMO:hIL6:HDEL*) and *in vitro* cleavage by His:bdSENP1 expressed in *Escherichia coli* to produce tag-less hIL6 (Islam et al., 2019a). For the current study, we generated a chimeric construct *BiP:sCBD:bdSUMO:hIL6:HDEL* (*BsCShIL6H*) (**Supplementary Figure S1A**). *BsCShIL6H* was transformed into *A. thaliana* protoplasts, either alone or together with *His:bdSENP1*, and protein extracts were analyzed by western blotting with anti-IL6 and anti-CBD antibodies. Both antibodies detected a protein band at 34 kDa, which corresponded with full-length BsCShIL6H expressed alone (**Supplementary Figures S1B, C**). By contrast, following the coexpression of *His:bdSENP1* with *BsCShIL6H*, anti-IL6-antibody detected a 21 kDa band, which matched the known size of hIL6 (**Supplementary Figure S1B**), whereas anti-CBD antibody detected a 13 kDa band that corresponded with the sCBD:bdSUMO fragment (**Supplementary Figure S1C**). Thus, bdSENP1 could act in the ER to cleave proteins containing the bdSUMO domain precisely.

### Design of Constructs for High-Level Expression of Recombinant hLIF and Transient Expression of Recombinant Proteins in *N. benthamiana*

To test further the effectiveness of removing foreign domains from the target protein *in vivo*, we designed a recombinant gene for expression of hLIF in *N. benthamiana* plants. For high-level expression of hLIF *in planta*, we incorporated the M domain, a highly glycosylated domain derived from human CD45 (Kang et al., 2018), to act as a translation enhancer domain (TED) at the N-terminus of hLIF to induce high levels of protein accumulation. Fusion of the M domain to a target protein causes an up to 6–8-fold increase in the accumulation of target protein in the ER *in planta* (Kang et al., 2018). To increase the solubility of the recombinant protein (Murashima et al., 2003), we included CBM3, a CBD from *Clostridium thermocellum*, next to the M domain (Islam et al., 2019a). We also tested the effect on protein expression of p38, a viral coat protein from *Turnip crinkle virus* (Qu et al., 2003); p38 is a gene silencing suppressor used when genes are transiently expressed in plants *via Agrobacterium tumefaciens*-mediated transformation (Qu et al., 2003; Thomas et al., 2003). As p38 accumulates to high levels in plants when used as a gene silencing suppressor, we speculated it might also act as a TED when fused to a target protein. To enable removal of N-terminal TED and CBM3 *via* bdSENP1-mediated cleavage in the ER *in vivo*, we fused the bdSUMO domain to the C-terminus of CBM3 or TED, respectively. In addition, we added a His×6 tag downstream from bdSUMO so that it could be used to purify recombinant hLIF from extracts of *N. benthamiana* leaves following *in vivo* cleavage of the recombinant protein. hLIF is a glycoprotein that has seven potential N-glycosylation sites (**Supplementary Figure S2**), thus causing the N-linked glycosylation of recombinant hLIF in the ER. The BiP leader sequence and an ER retention signal HDEL (Gomord et al., 1997) were added to the N and C-termini, respectively, of the recombinant constructs (**Figure 2A**), thus giving *BiP:M:CBM3:bdSUMO : His:hLIF : HDEL* (*MCS:hLIF*) and *BiP:p38:bdSUMO : His:hLIF : HDEL* (*p38:bdSUMO:hLIF*) (**Figure 2A**). A glycine and serine-rich (glycine-glycine-glycine-glycine-serine × 2) flexible linker (L) was inserted to prevent steric hindrance between the domains.

MCS:hLIF and p38:bdSUMO:hLIF were expressed in *N. benthamiana* leaves using *A. tumefaciens*-mediated transient expression (Marillonnet et al., 2005). Leaf tissue from 6-week-old plants was syringe-infiltrated with *A. tumefaciens* harboring *MCS:hLIF* or *p38:bdSUMO:hLIF*, together with *A. tumefaciens* harboring the *p1300-p38* construct (**Supplementary Figure S5**) (Qu et al., 2003). To determine the levels of protein expression, agroinfiltrated leaves were collected 3–5 days post infiltration (DPI), and total soluble proteins (TSPs) were extracted and analyzed using western blotting with anti-LIF antibody (**Figure 2B**). MCS:hLIF and p38:bdSUMO:hLIF-specific protein bands were detected at a range of molecular weights between 66 and 97 kDa. The full-length monomers of MCS:hLIF and p38:bdSUMO:hLIF were predicted to be 55 and 67 kDa, respectively. The multiple bands may represent recombinant proteins with different degrees of N-glycosylation. hLIF contains multiple N-glycosylation sites that display varying levels of N-glycosylation in animal cells; moreover, recombinant hLIF expressed in rice cells showed a variable glycosylation pattern (Youngblood et al., 2014). Blotting with anti-LIF antibody detected multiple protein bands ranging from 30 to 42 kDa (**Figure 2B**). These protein species represented fragments of MCS:hLIF and p38:bdSUMO:hLIF. From the observed sizes of the fragments, recombinant hLIF appeared to have been cleaved downstream of the bdSUMO domain; however, GFP or hIL6 fusion proteins containing the bdSUMO domain did not show any nonspecific cleavage in plants (Islam et al., 2019a; Islam et al., 2019b). The cause of *in vivo* degradation of MCS:hLIF and p38:bdSUMO:hLIF immediately adjacent to the bdSUMO domain was unclear. The expression level of p38:bdSUMO:hLIF was slightly higher than that of MCS:hLIF in *N. benthamiana* leaves (**Figure 2B**), and we accordingly selected p38:bdSUMO:hLIF for further study and to produce hLIF in plants.

To determine the optimum conditions for high-level expression of p38:bdSUMO:hLIF in *N. benthamiana* leaves, we examined its expression in leaf tissues at different time points after infiltration. TSP was extracted from leaves harvested at 3, 5, and 7 DPI and separated by SDS-PAGE, followed by Coomassie brilliant blue (CBB) staining (**Figure 2C**) and western blot analysis with anti-LIF antibody (**Figure 2D**). As observed previously (**Figure 2B**), multiple bands representing p38:bdSUMO:hLIF were detected in two different regions of the blot, between 66 and 97 kDa and between 30 and 42 kDa (**Figure 2D**). The intensity of the signal from both the larger and smaller regions increased with DPI, and was strongest in samples collected 7 DPI. Coexpression of the silencing suppressor p38 greatly enhanced expression of p38:bdSUMO:hLIF (Qu et al., 2003; Thomas et al., 2003).

### Release of His:hLIF From Recombinant Protein *via* bdSENP1-Mediated Proteolysis *In Planta*

**Figure 3:**
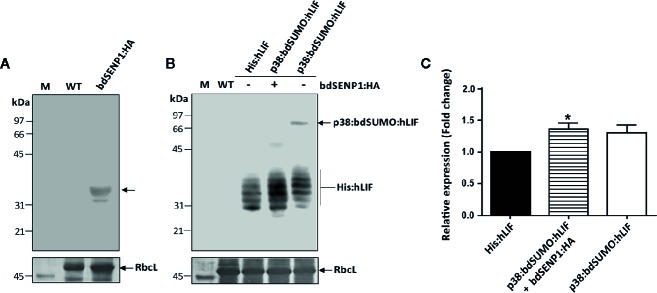
His:hLIF is released from recombinant protein containing the bdSUMO domain by bdSENP1-mediated proteolysis in the ER *in planta*. **(A**, **B)** *In vivo* removal of the additional N-terminal domains of His:hLIF fusion proteins following bdSENP1:HA coexpression. *N. benthamiana* leaves were infiltrated with a mixture of *A. tumefaciens* harboring *p1300-His:hLIF*, *p1300-p38:bdSUMO:hLIF,* and *p1300-p38*, or with (+) or without (−) the *p1300-bdSENP1:HA*. Total soluble proteins were prepared from a leaf tissue harvested at 7 DPI, separated using 12.5% SDS-PAGE, and analyzed by western blotting with anti-HA **(A)** or anti-LIF **(B)** antibodies. The membrane was subsequently stained with Coomassie brilliant blue (CBB). The large subunit of the rubisco complex (RbcL) was used as a loading control. M: molecular weight standard; WT: wild-type *N. benthamiana* leaf tissue extract. The arrows indicate the position of protein bands: bdSENP1:HA at 33 kDa; p38:bdSUMO:hLIF at 66 to 97 kDa; and His:hLIF at 30 to 42 kDa. **(C)** Comparison of expression levels of His:hLIF and p38:bdSUMO:hLIF. The signal intensity of protein bands in the image of western blot analysis in **(B)** was quantified using software supplied to LAS4000 image analyzer (Fujifilm, Japan); values are represented as the fold-change relative to the 7 DPI of His:hLIF. Three independent experiments were performed from three different plant leaves. Error bars, SEM (*n* = 3). Statistical analysis was performed by one-way analysis of variance (ANOVA) with Tukey’s *post-hoc* multiple comparison analysis; *P < 0.044.

We investigated whether bdSENP1:HA recognized the bdSUMO domain and cleaved the recombinant hLIF fusion protein (p38:bdSUMO:hLIF) in the ER *in planta*. We also examined the effect of p38 fusion on the expression of recombinant proteins in plants. As a control, we generated an *hLIF* construct without the p38 fusion (*His:hLIF*) (**Figure 2A**). *N. benthamiana* leaf cells were infiltrated with *A. tumefaciens* harboring the constructs *His:hLIF* alone, *p38:bdSUMO:hLIF* alone, or *p38:bdSUMO:hLIF* together with *bdSENP1:HA* (**Figure 2A**); for all three transformations, *A. tumefaciens* harboring the construct *p38* (**Supplementary Figure S5**) was also coinfiltrated to ensure high-level expression of both proteins. TSPs extracted from the leaves sampled on 7 DPI were analyzed by western blotting with anti-LIF antibody (**Figure 3B**). In the absence of *bdSENP1:HA* coexpression, anti-LIF antibody detected a band at approximately 66 to 97 kDa that corresponded with full-length p38:bdSUMO:hLIF; by contrast, hLIF-specific bands approximately 30 to 42 kDa in size were observed when *bdSENP1:HA* was coexpressed (**Figure 3B**), His:hLIF expressed alone was detected in the same size (30~42 kDa) (**Figure 3B**). Blotting with anti-HA antibody produced a 33 kDa band, corresponding with bdSENP1:HA, when bdSENP1:HA was coexpressed with hLIF recombinant protein (**Figure 3A**). bdSENP1:HA, when coexpressed with p38:bdSUMO:hLIF *in planta*, is thus able to recognize the bdSUMO domain of the recombinant protein and to cut at its cleavage site, thereby releasing the C-terminal His:hLIF.

We examined the cleavage by bdSENP1:HA *in vivo*. We performed N-terminal amino acid sequencing of His:hLIF. The multiple bands between 30 and 42 kDa were excised from the PVDF membrane after SDS-PAGE electroblots and subjected to Edman degradation. We found that the N-terminal 5 aa residues are H-H-H-H-H (**Supplementary Figure S4**), indicating that all these multiple bands to be His:hLIF with the same His×5. Moreover, this result suggests that bdSENP1 cleaves the exact recognition site at the C-terminus of bdSUMO moiety in the ER *in planta*.

Next, we examined whether p38 fusion enhances the protein level in plants. Densitometry analysis was performed to compare the intensity of the signals detected by anti-LIF antibody from TSPs of plant tissues expressing *His:hLIF* alone, *p38:bdSUMO:hLIF* alone, or *p38:bdSUMO:hLIF* together with bdSENP1:HA. Following the coexpression of bdSENP1:HA with p38:bdSUMO:hLIF, the level of released His:hLIF expression increased 1.3-fold to compare with His:hLIF alone (**Figure 3C**), confirming that p38 fusion enhances the expression level of a target protein.

### Production of His:hLIF From *N. benthamiana* Leaf Tissues *via* Ni^2+^–NTA Affinity Purification

**Figure 4:**
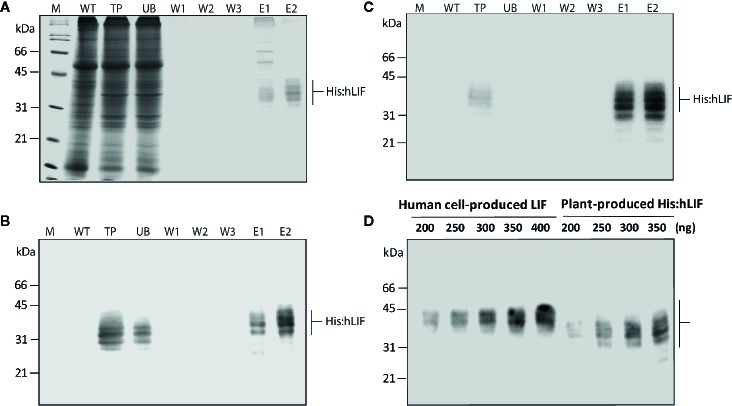
Production of His:hLIF from *N. benthamiana* leaf tissue extracts by Ni^2+^–NTA affinity column chromatography. **(A)** Ni^2+^–NTA affinity purification of His:hLIF. Total soluble proteins were extracted from agroinfiltrated *N. benthamiana* leaf tissue, and recombinant His:hLIF was purified using a Ni^2+^–NTA agarose column with 250 mM imidazole solution as eluent (E). The fractions obtained from purification were analyzed using 12.5% SDS-PAGE followed by Coomassie brilliant blue (CBB) staining. **(B, C)** Western blot analysis of purified His:hLIF. The His:hLIF elution fractions were separated using 12.5% SDS-PAGE and analyzed by western blotting with anti-LIF **(B)** or anti-His **(C)** antibodies. M, molecular weight standard; WT, wild-type *N. benthamiana* leaf tissue extracts; UB, unbound fraction; W, wash-off solution. The arrows indicate the positions of His:hLIF protein bands at 30 to 42 kDa. **(D)** Quantification of His:hLIF produced in plants. The concentration of purified His:hLIF was determined using the Bradford protein assay. Samples containing 200 to 350 ng His:hLIF produced *in planta* or commercial LIF produced in a human cell line were separated using 12.5% SDS-PAGE, and the amounts of each protein were compared using signal intensity from a western blot analysis with anti-hLIF antibody. The concentration of the commercial LIF was provided by the manufacturer. Protein molecular weight standards are marked at the left-hand side.

Following bdSENP1-mediated proteolytic cleavage of the full-length hLIF recombinant protein (p38:bdSUMO:hLIF) *in planta*, we purified His:hLIF from plant extracts. Agroinfiltrated leaves were harvested at 7 DPI, and TSPs were prepared from 40 g samples of fresh leaf tissue. His:hLIF was purified by Ni^2+^–NTA affinity column chromatography using 250 mM imidazole as the eluent. The purity of His:hLIF was examined by SDS-PAGE followed by CBB-staining (**Figure 4A**). The CBB-stained gel exhibited multiple bands of His:hLIF between 30 and 42 kDa. No other bands were detected in the second elution fraction, indicating a high degree of purity. The purified His:hLIF protein was confirmed by western blotting with anti-LIF (**Figure 4B**) and anti-His antibodies (**Figure 4C**). These results confirmed that our *in vivo* system employing the bdSUMO domain and bdSENP1 to remove foreign domains was highly effective in plant cells at removing domains fused to the N-terminus of a target protein.

**Figure 5:**
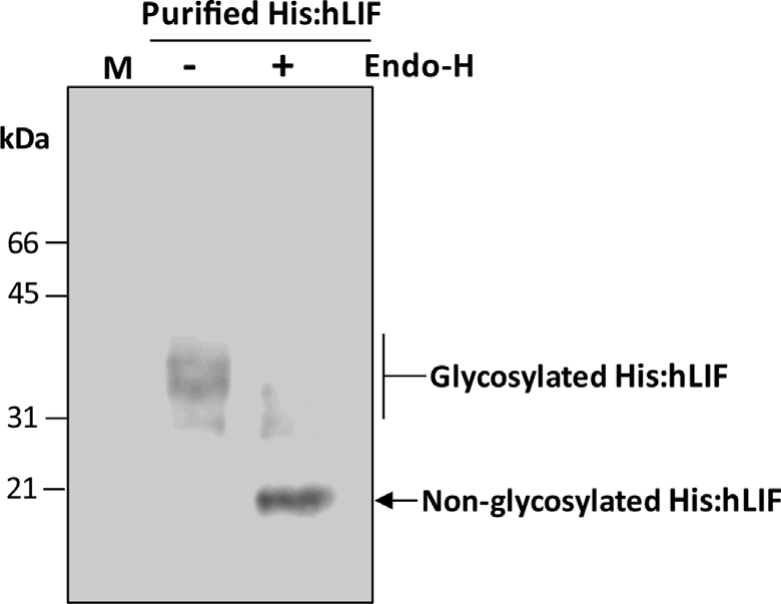
His:hLIF produced *in planta* exhibits different degrees of N-glycosylation. Purified His:hLIF was treated with (+) or without (−) endoglycosidase H (Endo-H), and the migration patterns of the treated proteins were analyzed by western blotting with anti-LIF antibody. The arrows indicate N-glycosylated His:hLIF protein bands at 30 to 42 kDa and deglycosylated His:hLIF protein bands at 20 kDa. M, molecular weight standard.

Purified His:hLIF was quantified by the Bradford method, using bovine serum albumin (BSA) as a standard (Bradford, 1976). To confirm this method of protein quantification, we used western blot analysis with the anti-LIF antibody (**Figure 4D**) and densitometry analysis (**Supplementary Figure S3**) to compare the intensity of the signals detected from His:hLIF produced by plants and commercial hLIF (Cat. No. 14890-H08H) produced in a human cell line (HEK293 cells). The two proteins showed a similar band intensity that matched the estimated concentrations, confirming the accuracy of our estimate of His:hLIF protein concentration. The yield of His:hLIF from production in plants was 32.49 mg/kg fresh leaves weight (FW) with approximately > 95% purity. hLIF produced in human cells was, however, detected at a slightly higher position than that produced in plants (30 to 42 kDa). The underlying cause of this difference was unknown. Nevertheless, the slightly higher molecular weight of commercial hLIF can be explained by the difference in the type of N-glycans. Commercial hLIF was produced in the HEK293 cell suspension system through the secretory pathway. Thus, it is likely that hLIF has a complex type of N-glycans *via* modification in the Golgi apparatus before secretion in the media (Ren et al., 2019). However, the plant-produced His:hLIF should have high mannose type N-glycan because it was retained in the ER *via* ER retention signal (see **Figure 5**).

### Recombinant His:hLIF Produced in Plants Has High Mannose-Type N-Glycans

We next examined the nature of the multiple bands observed in plant-produced His:hLIF. hLIF has seven N-glycosylation sites: Asp-9, Asp-34, Asp-63, Asp-73, Asp-96, Asp-105, and Asp-116 (**Supplementary Figure S2**). It was possible that not all these sites were fully N-glycosylated *in planta*, thereby resulting in multiple bands. To test this hypothesis, His:hLIF was treated with endoglycosidase H (Endo-H) as we reasoned that, since hLIF fusion protein contained the ER retention signal HDEL at its C-terminus, the N-glycans were likely to be the high mannose type and thus sensitive to Endo-H (Islam et al., 2019a). Endo-H-treated His:hLIF was analyzed by western blotting with anti-LIF antibody (**Figure 5**). Following Endo-H treatment, the intensity of the multiple His:hLIF bands observed between 30 and 42 kDa was greatly diminished and a new protein band appeared at 20 kDa, the predicted size of His:hLIF. This result and the N-terminal amino acid sequencing analysis (**Supplementary Figure S4**) indicate that the original multiple bands were forms of His:hLIF with varying degrees of N-glycosylation. It thus appears that His:hLIF is N-glycosylated to different levels in plants, as has been observed with hLIF produced in animal cells. Furthermore, the N-glycosylation pattern of His:hLIF confirms that it is localized to the ER.

### His:hLIF Produced in Plants Contains Low Levels of Endotoxin and Can Maintain mESC Pluripotency

Endotoxin contamination is a critical concern for *in vitro* culture of ESCs whenever growth factors or cytokines produced in *E. coli* are used. Studies show that stem cells exposed to >1 EU/ml endotoxin exhibit morphological alterations and significantly reduced rates of proliferation (Nomura et al., 2017). As we had used *A. tumefaciens* to induce transient expression of His:hLIF in plants, we examined endotoxin contamination in His:hLIF (phLIF) produced in plants using the chromogenic *Limulus* amebocyte lysate (LAL) assay. This indicated that phLIF contained <0.61 EU/µg (< 0.06 ng/µg) endotoxin, which was beneath the accepted safety limit [1 EU/µg (< 0.1 ng/µg)] (Magnusdottir et al., 2013). Despite the use of *A. tumefaciens* to deliver the recombinant gene construct to plant cells, endotoxin levels in the final product were thus very low (Islam et al., 2019a).

**Figure 6:**
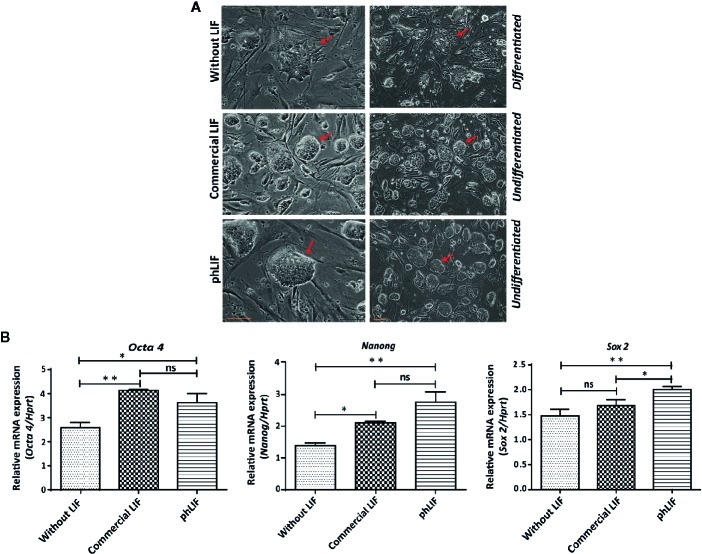
His:hLIF produced *in planta* is biologically active. **(A)** Biological activity of phLIF to maintain the pluripotency of mouse embryonic stem cells (mESCs). mESCs were cultured in the presence of 10 ng/ml phLIF or commercial LIF for four passages; mESCs without LIF supplements were included as a negative control. Cellular morphology was observed using phase-contrast microscopy. Scale bars = 100 µm. **(B)** Transcript levels of pluripotency marker genes in mESCs cultured with phLIF. mESCs were cultured in the presence of phLIF (10 ng/ml) or commercial LIF (10 ng/ml), or without LIF for four passages. Total RNA was extracted from each culture, and the transcript levels of *Oct4*, *Nanog*, and *Sox2* were determined using qRT-PCR; the housekeeping gene, *Hprt*, was used as an endogenous control. The data were collected from six independent experiments, and the technical replicates were repeated three times. Data represent relative mRNA levels after normalization. Error bars, SEM (*n* = 6). Statistical analysis was performed by one-way analysis of variance (ANOVA) with Tukey’s *post-hoc* multiple comparison analysis (GraphPad Prism 6); *P < 0.010; **P < 0.0019; ns, no significant difference.

We next determined whether phLIF was biologically active. LIF is a crucial regulator that maintains pluripotency in human and mouse embryonic stem cells (mESCs) by suppressing stem cell differentiation (Shabbir et al., 2010; Hirai et al., 2011). We examined growth and pluripotency of mESCs in the presence of phLIF, using commercial LIF produced in *E. coli* as a PC. As elimination of LIF from the culture medium results in the rapid differentiation of mESCs (Shabbir et al., 2010), we also cultured mESCs in the absence of hLIF as a negative control. mESCs were cultured with 10 ng/ml of phLIF or commercial LIF for four passages, and cellular morphology was observed using phase-contrast microscopy. There were no apparent differences between the two LIF treatments; mESCs treated with either phLIF or commercial LIF appeared similar, and both showed spherical cellular morphology, a characteristic feature of undifferentiated status and pluripotency (**Figure 6A**). By contrast, mESCs cultured without LIF supplement showed spontaneous cellular differentiation (**Figure 6A**). phLIF thus appeared to be active, with a similar effect to commercial LIF. To further confirm phLIF activity, we analyzed mRNA levels of the mouse transcription factors, *Oct4*, *Sox2*, and *Nanog*, which are pluripotency markers (Theunissen & Jaenisch, 2017). mESCs cultured in the presence of phLIF or commercial LIF showed significantly elevated expression of all three genes compared to those cultured without LIF (**Figure 6B**). Although mRNA levels of *Oct* and *Nanog* were slightly higher in cells cultured with phLIF than with commercial hLIF, the differences were statistically insignificant (**Figure 6B**). phLIF was thus able to maintain the undifferentiated state of mESCs to a similar degree as the commercial product.

## Discussion

We have described a protocol employing the SUMO domain and SUMO-specific protease bdSENP1 for the *in vivo* removal of foreign domains fused to a target protein. The highly active bdSENP1 proteolytically cleaved recombinant proteins containing the bdSUMO domain in the ER *in planta*. The protocol involved two steps: initially, a chimeric recombinant target protein was engineered that included the bdSUMO domain fused to its N-terminal region. bdSENP1 was then coexpressed with the recombinant target protein. Finally, we demonstrated that we could use this protocol to produce hLIF without N-terminal foreign domains in *N. benthamiana* leaf cells.

SUMO proteases are cytosolic proteins (Bailey and O’Hare, 2004; Hickey et al., 2012). bdSENP1 produced by *E. coli* has been used previously to remove domains fused to the N-terminus of a target protein following immobilization on cellulose beads *in vitro* (Islam et al., 2019a). In most cases, however, researchers have expressed recombinant proteins in the ER or chloroplasts of plants. We therefore confirmed that bdSENP1 could act in the ER by demonstrating proteolytic cleavage in that organelle of recombinant proteins containing bdSUMO domains expressed in both *A. thaliana* protoplasts and intact *N. benthamiana* leaf cells.

The advantage of this approach is that many different foreign domains may be fused to a target protein to increase its expression level or solubility and then be removed after expression *in vivo*, thereby allowing production of a target protein without any extra foreign domains. This strategy has been widely used for the production of many endogenous proteins, including growth factors and cytokines in animal cells as well as proteases such as pepsin and trypsin (Shi et al., 2011; Kim et al., 2011; Shen et al., 2017). These proteins are expressed as preproproteins and then converted to their functional form *via* proteolytic processing. We employed a similar strategy to produce a target protein without foreign functional domains. Since the foreign domains were proteolytically removed, many domains with a variety of useful functions could be fused to the target protein. Transcription or translation levels can be enhanced by fusion of certain domains (Kang et al., 2018; Alqazlan et al., 2019). Although such domains may be beneficial for high-level expression or to increase the solubility of target proteins, their retention on the final protein is not desirable as they may interfere with its function. Moreover, since bdSENP1-mediated proteolysis occurred *in vivo,* there was no need to prepare the protease separately and thus no need for an extra step, unlike the use of *in vitro* proteolysis to purify a target protein.

Many domains have been identified that enhance protein production levels when fused to a target protein (Conley et al., 2011; Kang et al., 2018; Alqazlan et al., 2019). We examined whether fusion of the gene silencing suppressor p38 to the target protein increased protein levels. p38, the *Turnip crinkle virus* coat protein, is often coexpressed with a target protein to suppress gene silencing suppressors and greatly improves expression levels of heterologous genes (Qu et al., 2003; Thomas et al., 2003; Islam et al., 2019a); in addition, p38 itself accumulates to high levels in the plant cytosol. Many viral proteins are expressed at high levels and show a high degree of stability in plants (Dennis et al., 2018; Margolin et al., 2018). When we tested whether p38 induced accumulation of fusion proteins in the ER, we found that p38 fusion to the hLIF increased recombinant protein levels by 1.3-folds in plants; in fact, p38 increased protein expression to a greater extent than the M domain, which was shown to increase accumulation of ER-targeted proteins significantly (6- to 8-fold increase in level) in plants (Kang et al., 2018). Although fusion of p38 to a target protein improved protein production in plants, we did not address the underlying processes responsible, and further study is required to elucidate the mechanism.

We demonstrated the utility of this system by producing a target protein, hLIF, using *A. tumefaciens*-mediated transient expression in *N. benthamiana* leaf tissues. In the first step, we engineered a high-level expression vector that included viral coat protein p38 as a TED fused to the N-terminus of bdSUMO followed by the target protein hLIF. The fusion protein, p38:bdSUMO:hLIF, was transiently expressed at high levels *in planta*. It was noted that p38:bdSUMO:hLIF was broken downstream of the bdSUMO domain. Other bdSUMO domain-containing fusion constructs, including *BsCSGH*, *BsCShIL6H*, *MCS : LysP11* (Islam et al., 2019b), and *MCS-hIL6* (Islam et al., 2019a), did not show endogenous proteolytic cleavage at the C-terminal region of the bdSUMO domain in the ER; however, such degradation appeared to be specific to the recombinant protein containing hLIF. The full-length p38:bdSUMO:hLIF was efficiently cleaved when coexpressed with bdSENP1. The His×6 tag positioned immediately after the bdSENP1 cleavage site in the recombinant protein was used for affinity purification of proteins produced in plant cell extracts. This approach produced His:hLIF at a yield of approximately 32.49 µg/g FW leaf tissue at near homogeneity (Nausch et al., 2012; Sabalza et al., 2014; Islam et al., 2018; Islam et al., 2019a).

It is essential to investigate whether recombinant protein produced in heterologous expression systems such as plants are biologically active. ESCs are characterized by three unique features: pluripotency, self-renewal, and unlimited proliferation. LIF is one of the regulatory cytokines involved in maintaining these features in mESCs (Shabbir et al., 2010; Hirai et al., 2011). The ability of plant-produced His:hLIF (phLIF) to maintain the undifferentiated “näive state” (pluripotency) of mESCs was tested and found to be nearly equivalent to commercial LIF produced in *E. coli*. Although phLIF retained an N-terminal histidine tag (His×6), the tag did not appear to affect its biological activity, consistent with previous studies that found no substantial adverse effects of this tag on *in vitro* biological activity of many proteins or on the ability of hLIF to maintain pluripotency in mESCs (Huyton et al., 2007; Imsoonthornruksa et al., 2011; Taheri et al., 2018).

In conclusion, we developed a new strategy that used the bdSUMO domain/bdSENP1 protease to remove foreign domains from a target protein following the production of recombinant protein *in planta*. The bdSUMO domain-containing recombinant protein was coexpressed with the highly active bdSUMO domain-specific protease bdSENP1. bdSENP1 proteolytically removed the N-terminal domains together with the bdSUMO domain from the recombinant proteins in the ER, thereby releasing the target protein from the recombinant protein *in planta*. We demonstrated that hLIF produced *in planta* was biologically active. This method offers considerable potential for developing plant platforms capable of producing biologically active proteins without foreign domains.

## Data Availability Statement

All datasets analyzed for this study are included in the article/**Supplementary Material**.

## Author Contributions****

RI and IH designed the research. RI performed most of the experiments. SC and TM participated in biological activity. KS participated in project planning and provided technical assistance for the biological activity. RI and IH interpreted the results and wrote the manuscript.

## Funding

This work was supported by the Technology Innovation Program (No. 10063301, Industry core technology development of plant-derived biomaterials for the stem cell culture medium) funded By the Ministry of Trade, Industry & Energy (MOTIE, Korea) and Korea Research Fellowship Program through the National Research Foundation of Korea (NRF) funded By the Ministry of Science and ICT (No. 2016H1D3A1938045), Korea.

## Conflict of Interest

The authors declare that the research was conducted in the absence of any commercial or financial relationships that could be construed as a potential conflict of interest.
