# Supplementary material for: In Vivo Removal of N-Terminal Fusion Domains From Recombinant Target Proteins Produced in Nicotiana benthamiana
Source: Front Plant Sci. 2020 Apr 8;11:440. doi: 10.3389/fpls.2020.00440 (PMC7160244; doi:10.3389/fpls.2020.00440)
Supplement: Supplementary file 1 [file DataSheet_1.docx]

**Supplementary** **Material**

***In Vivo* Removal of N-Terminal Fusion Domains** **from Recombinant Target Proteins Produced in *Nicotiana benthamiana***

Md Reyazul Islam^1^, Seoyoung Choi^2^, Thangarasu Muthamilselvan^1^, Kunyoo Shin^2^, and Inhwan Hwang^1,2^*****

^1^Division of Integrative Biosciences and Biotechnology, Pohang University of Science and Technology, Pohang, 37673, South Korea.

^2^Department of Life Sciences, Pohang University of Science and Technology, Pohang, 37673, South Korea.

***Correspondence:** Inhwan Hwang

Division of Integrative Biosciences and Biotechnology, Pohang University of Science and Technology, Pohang, 37673, South Korea.

Tel: +82-54-279-2128

E-mail: ihhwang@postech.ac.kr


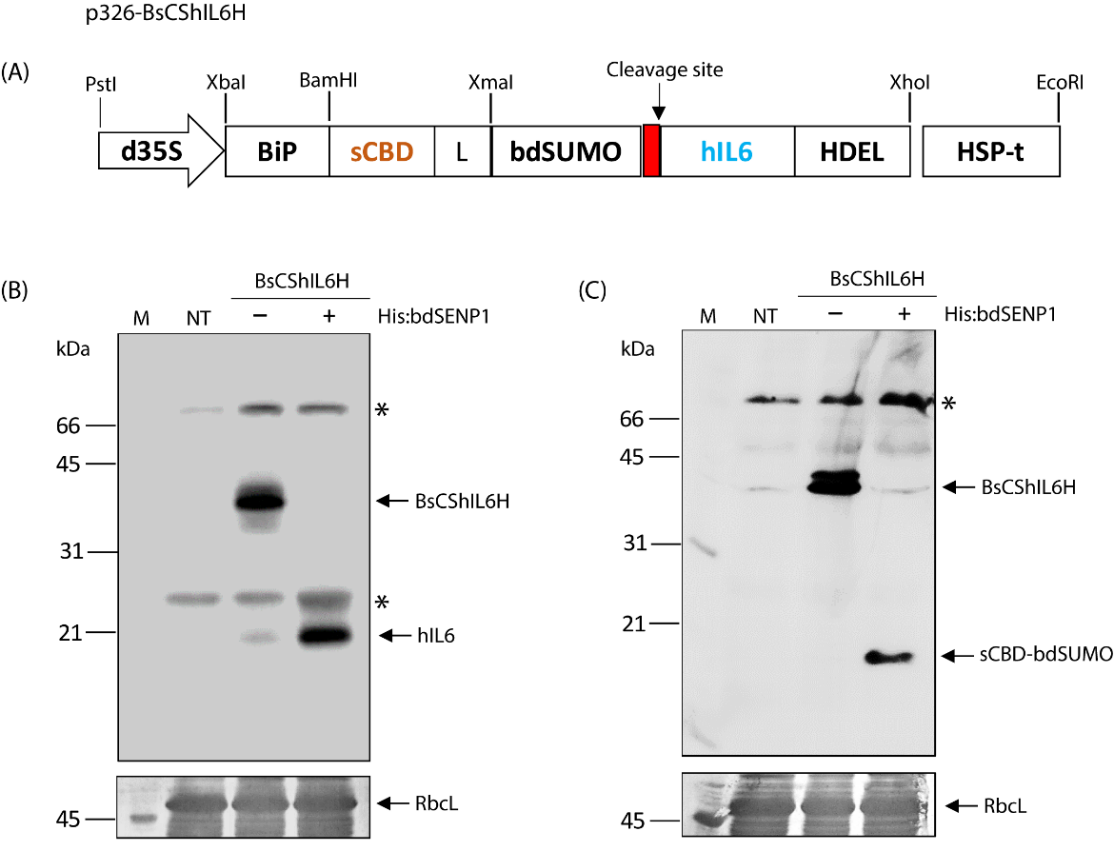


**Supplementary Figure S1. *In vivo* cleavage of fusion proteins containing the bdSUMO domain following co-expression of bdSENP1 in the ER of *Arabidopsis thaliana* protoplasts.**

(**A**) Schematic representation of the chimeric construct used in *Arabidopsis thaliana* protoplasts. The expression vector, p326-BsCShIL6H, consists of sCBD, a plant codon-optimized cellulose-binding domain (sCBD) derived from *Trichoderma reesei*; bdSUMO, a small ubiquitin-related modifier from *Brachypodium distachyon* (XP_003564931.1); bdSENP1, a bdSUMO-specific protease (XP_003567671.1) from *Brachypodium distachyon*; and hIL6, human interleukin-6. The expression construct was under the control of the cauliflower mosaic virus (CaMV) 35S promoter with the double-enhanced element (d35S) and the HSP terminator (HSP-t) from *A. thaliana*. The ER leader sequence, BiP, and an ER retention signal, HDEL, were fused to the 5ʹ and 3ʹ ends of the chimeric constructs, respectively.

(**B,C**) *In vivo* cleavage of the fusion protein containing bdSUMO domain following co-expression of His:bdSENP1 in the ER. *A. thaliana* protoplasts were transfected with either p326-BsCShIL6H alone (-) or with BsCShIL6H and p326-His:bdSENP1 (+). Total soluble proteins were extracted from transfected and non-transfected (NT) cells and analyzed by western blotting with anti-IL6 antibody (B) or anti-CBD antibody (C). The membranes used for western blot analysis were subsequently stained with Coomassie brilliant blue (CBB). The large subunit of the rubisco complex (RbcL) was used as a loading control. M: molecular weight standard. The arrows indicate the position of protein bands: BsCShIL6H at 34 kDa; hIL6 at 21 kDa; and sCBD:bdSUMO at 13 kDa. Asterisks indicate non-specific bands.

(A)


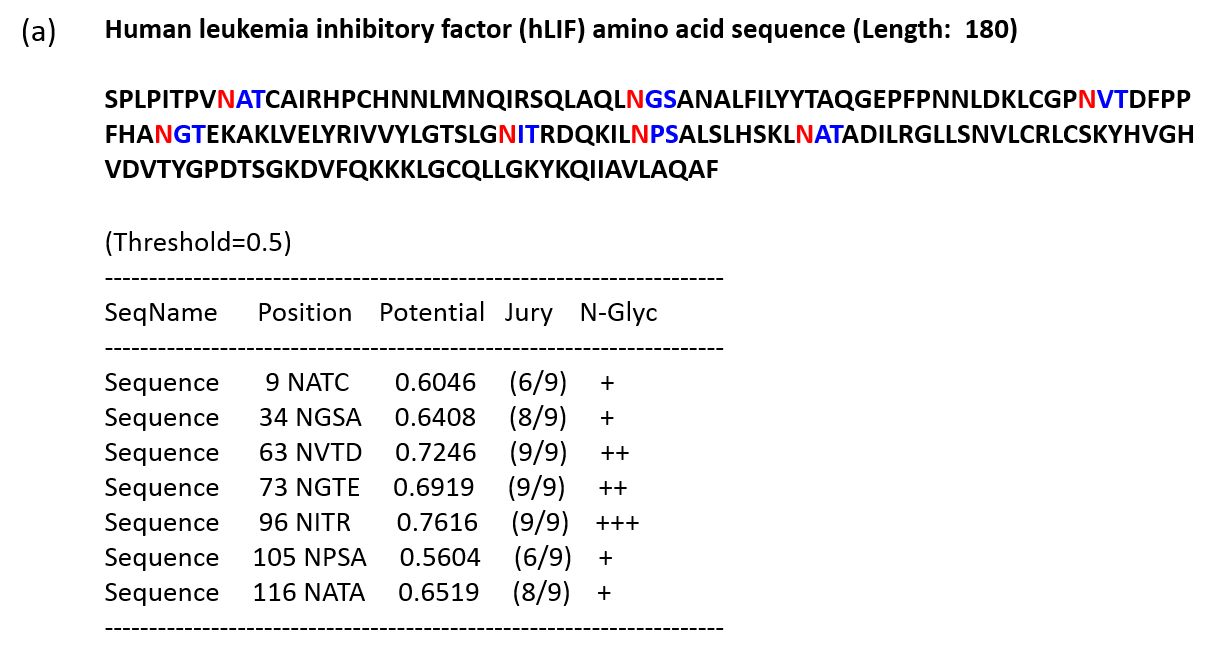


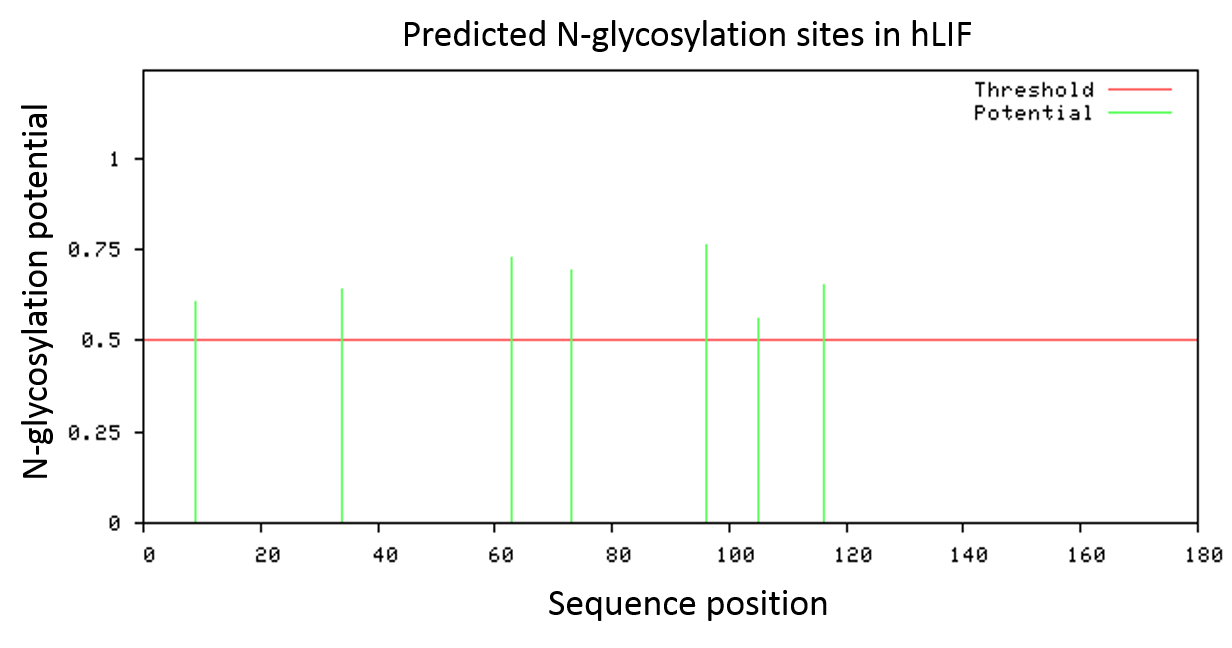
 (B)

**Supplementary Figure S2. Prediction of N-glycosylation sites in hLIF.** The amino acid sequence of hLIF (A) was analyzed for the presence of putative N-glycosylation sites using the online prediction tool of the **NetNGlyc** site (<http://www.cbs.dtu.dk/services/NetNGlyc/>). The **NetNglyc** online prediction tool calculates N-glycosylation sites in proteins using the consensus sequence context of Asn-Xaa-Ser/Thr (N-Xaa-S/T). The position of potential N-glycosylation sites of hLIF (Asp-9, Asp-34, Asp-63, Asp-73, Asp-96, Asp-105 and Asp-116) are indicated in green lines (B).


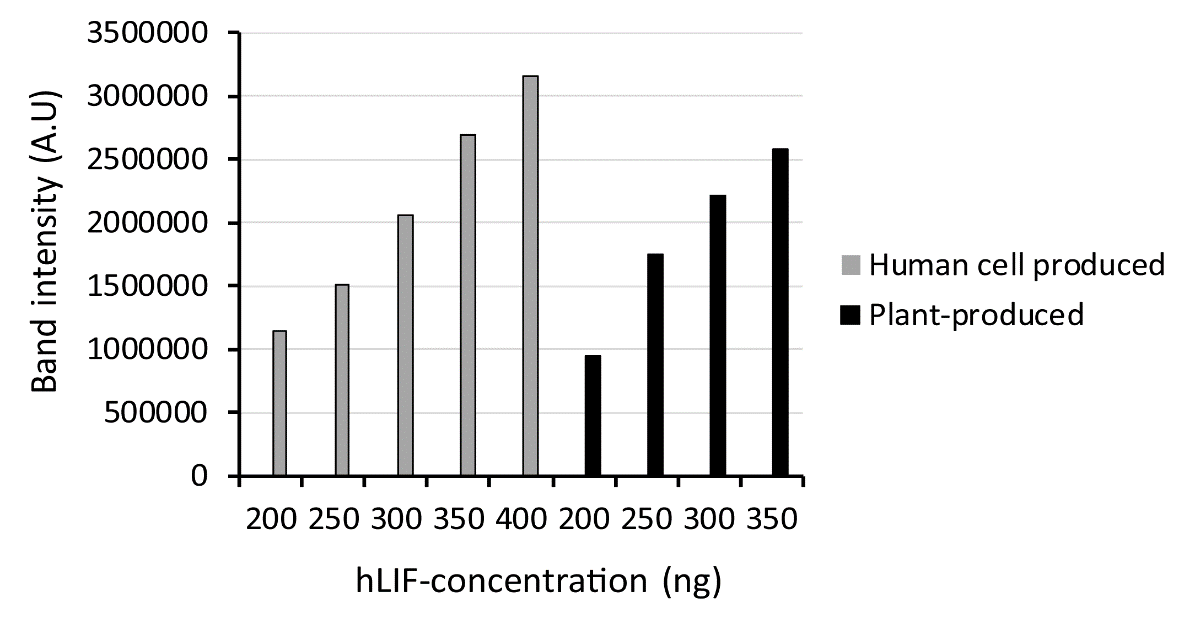


**Supplementary Figure S3. Densitometry analysis of purified His:hLIF obtained from *N. benthamiana* leaf tissues.** The chemiluminescence signal intensity of the plant-produced His:hLIF and human cell produced commercial LIF protein obtained by the western blotting using anti-LIF antibody (**Figure 4D**) were measured using Multi Gauge V2.2 densitometric software (Fujifilm, Japan), which determines the quantity of His:hLIF in arbitrary units (A.U.).


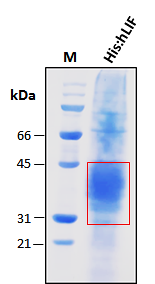
**(A)**

**(B)**


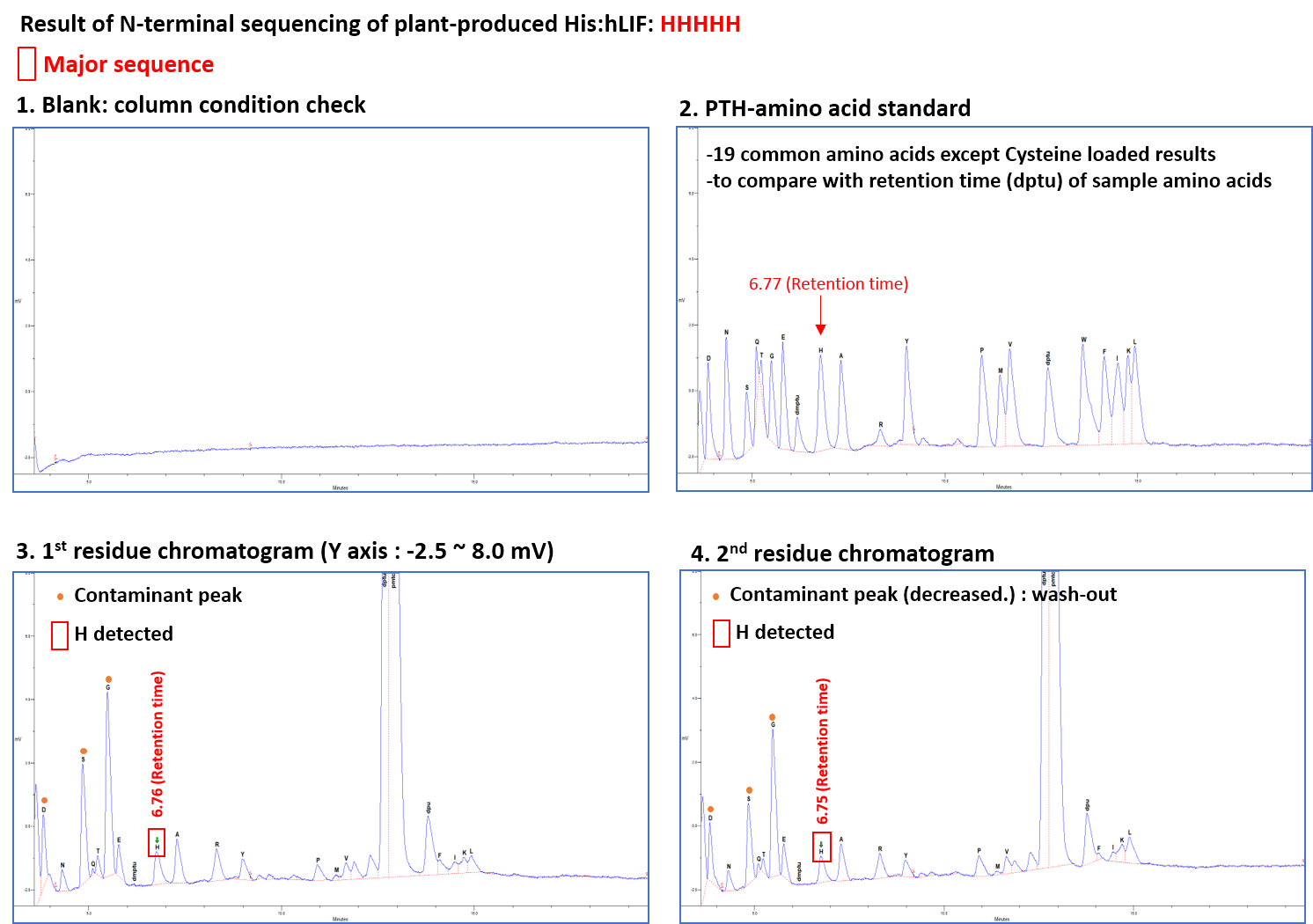


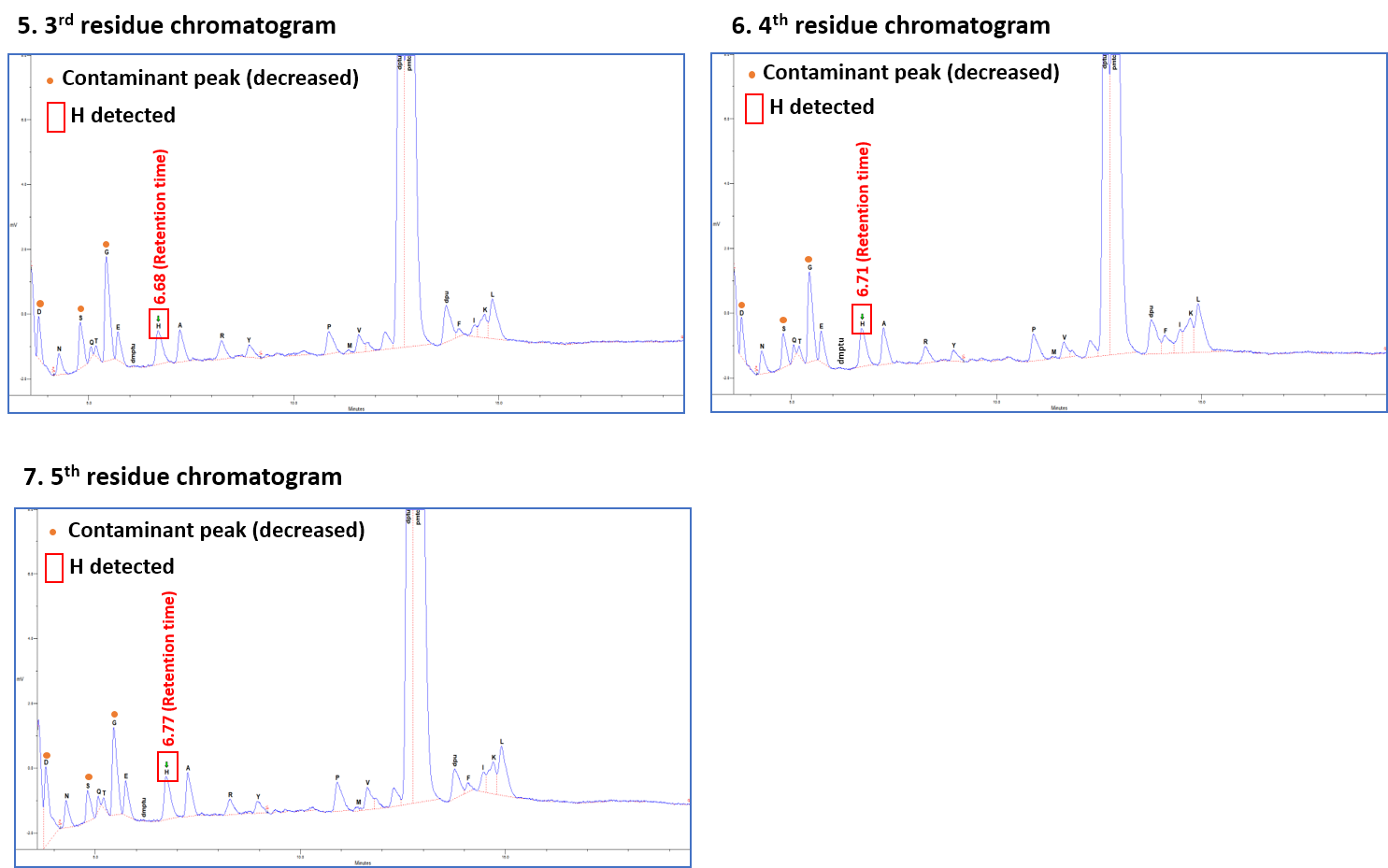


**Supplementary** **Figure S4. Determination of the N-terminal amino acid sequences of plant-produced His:hLIF.** Purified His:hLIF was separated *via* 12% SDS-PAGE under the reducing condition and electroblotted onto polyvinylidene difluoride (PVDF) membrane (Millipore, Billerica, MA, USA). The PVDF membrane was then stained with 0.02% Coomassie Blue R-250 until protein bands were visualized clearly (A). Proteins between 30 ~ 42 kDa bounds to PVDF membrane were excised and subjected to Edman degradation using an ABI 492 Procise Protein Sequencer System (Applied Biosystems, USA) (B). 1. column condition check (blank). Cycles 2: shows a chromatogram for PTH-amino acid standard. Cycles 3-7: sample amino acids of detected Histidine (H) chromatograms are showed (H detected in red boxes) to compare with retention time (dptu) of PTH-amino acid standard. dmptu and dptu are reaction byproducts of Edman degradation.


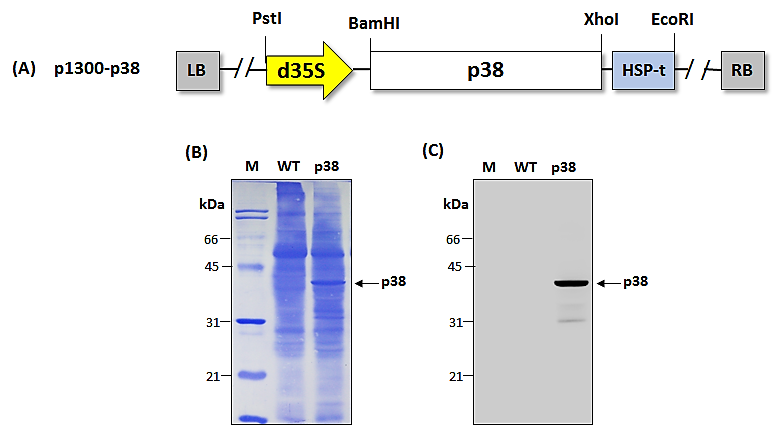


**Supplementary** **Figure S5. Schematic representation of the binary vector p1300-p38 of the *Turnip crinkle virus* silencing suppressor and transient expression in *N. benthamiana* leaf tissues.** (A) The expression vector, p1300-p38, was under the control of the cauliflower mosaic virus (CaMV) 35S promoter with the double-enhanced element (d35S) and the HSP terminator (HSP-t) from *A. thaliana*. LB: left border; RB: right border. (B-C) western blot analysis of p38 expression. *N. benthamiana* leaf tissue was harvested 7 days post-infiltration (DPI) with *A. tumefaciens-*infiltration of harboring p1300-p38. Total extracts were prepared and analyzed by western blotting with anti-p38 antibody (C). The membrane was subsequently stained with Coomassie brilliant blue (CBB) (B). M, protein molecular weight standards; WT, wild-type *N. benthamiana* leaf tissue extracts. The arrow indicates the position of the p38 protein bands (38 kDa).

**Supplementary** **Table S1. The nucleotide sequences of primers used in this study.**

| **Primer** | **Sequence 5’- 3’** |
| --- | --- |
| sCF | CGCGGATCCCTACCCAGAGTC |
| suR | CTTCTCCTTTACTCATCCCCCGGGGAGTTCCTGCACCACCAG |
| GF | CTGGTGGTGCAGGAACTCCCCGGGGGATGAGTAAAGGAGAAG |
| GR | CCGCTCGAGTTAGAGCTCATCTTTGTATAGTTC |
| sCF | CGCGGATCCCTACCCAGAGTC |
| suILR | CTCTCCTGGAGGAACCATACCACCAGTCTGATGTAAC |
| ILF | GTTACATCAGACTGGTGGTATGGTTCCTCCAGGAGAG |
| ILR | CCGCTCGAGCTAGAGCTCATCGTGCATCTGCCTAAGAGCCCT |
| HisSF-1 | CGCGGATCCATCACCACCATC |
| SR-2 | CCGCTCGAGCTACAACTCATCATGTCC |
| SF-3 | CGCGGATCCATCCATTCGTTCCGC |
| SHaR-4 | CCGCTCGAGCTACAACTCATCATGAGCGTAATCTGGAACATCGTATGGGTATCCAGCCTTCAAATCAAGTATC |
| csLF-1 | CGCGGATCCCGATGGCAAACATC |
| csLR-2 | GATGGGGAGGGGGCTCATGTGATGGTGGTGATGGTGACCACCAGTCTGATGTAAC |
| csLF-3 | GTTACATCAGACTGGTGGTCACCATCACCACCATCACATGAGCCCCCTCCCCATC |
| csLR-4 | CCGCTCGAGCTAGAGCTCATCGTGGAAGGCCTGGGCCAACAC |
| PF-1 | CGCGGATCCCGATGGAAAATGATCCTAG |
| PR-2 | TCCCCCCGGGCAATTCTGAGTGCTTGCCAT |

**Supplementary** **Table S2. The nucleotide sequences of primers used for quantitative RT-PCR in this study.**

| **Primer** | **Sequence 5’- 3’** |
| --- | --- |
| Hprt F | CTTCCTCCTCAGACCGCTTT |
| Hprt R | CATCATCGCTAATCACGACGC |
| Sox2 F | CAAAAACCGTGATGCCGACT |
| Sox2 R | CGCCCTCAGGTTTTCTCTGT |
| Oct4 F | CGTTCTCTTTGGAAAGGTGTC |
| Oct4 R | GAACCATACTCGAACCACATCC |
| Nanog F | AGGGTCTGCTACTGAGATGCTCTG |
| Nanog R | CAACCACTGGTTTTTCTGCCACCG |
